# Genome-Wide Transcriptional and Functional Analysis of Human T Lymphocytes Treated with Benzo[*α*]pyrene

**DOI:** 10.3390/ijms19113626

**Published:** 2018-11-17

**Authors:** Marie Liamin, Hélène Le Mentec, Bertrand Evrard, Laurence Huc, Frédéric Chalmel, Elisa Boutet-Robinet, Eric Le Ferrec, Lydie Sparfel

**Affiliations:** 1 Institut National de la Santé et de la Recherche Médicale (INSERM), Institut de Recherche en Santé, Environnement et Travail (IRSET-INSERM UMR 1085), 35000 Rennes, France; marie.liamin@univ-rennes1.fr (M.L.); helene.le-mentec@univ-rennes1.fr (H.L.M.); bertrand.evrard@inserm.fr (B.E.); frederic.chalmel@univ-rennes1.fr (F.C.); eric.leferrec@univ-rennes1.fr (E.L.F.); 2 Université de Rennes 1, Faculté des Sciences Pharmaceutiques et Biologiques, Structure Fédérative de Recherche Biosit UMS CNRS 3480/US INSERM 018, 35043 Rennes, France; 3 Toxalim (Research Centre in Food Toxicology), Université de Toulouse, INRA, ENVT, INP-Purpan, UPS, 31027 Toulouse, France; laurence.huc@inra.fr (L.H.); elisa.boutet@univ-tlse3.fr (E.B.-R.)

**Keywords:** benzo[*α*]pyrene, T lymphocytes, microarrays, migration, immunotoxicity

## Abstract

Polycyclic aromatic hydrocarbons (PAHs) are widely distributed environmental contaminants, known to affect T lymphocytes. However, the molecular targets and pathways involved in their immunotoxic effects in human T lymphocytes remain unknown. Here, we analyzed the gene expression profile of primary human T lymphocytes treated with the prototypical PAH, benzo[*α*]pyrene (B[*α*]P), using a microarray-based transcriptome analysis. After a 48 h exposure to B[*α*]P, we identified 158 genes differentially expressed in T lymphocytes, including not only genes well-known to be affected by PAHs such as the cytochromes P450 (*CYP*) *1A1* and *1B1*, but also others not previously shown to be targeted by B[*α*]P such as genes encoding the gap junction beta (*GJB*)-*2* and *6* proteins. Functional enrichment analysis revealed that these candidates were significantly associated with the aryl hydrocarbon (AhR) and interferon (IFN) signaling pathways; a marked alteration in T lymphocyte recruitment was also observed. Using functional tests in transwell migration experiments, B[*α*]P was then shown to significantly decrease the chemokine (C-X-C motif) ligand 12-induced chemotaxis and transendothelial migration of T lymphocytes. In total, this study opens the way to unsuspected responsive pathway of interest, i.e., T lymphocyte migration, thus providing a more thorough understanding of the molecular basis of the immunotoxicity of PAHs.

## 1. Introduction

Benzo[*α*]pyrene (B[*α*]P) is a prototypical polycyclic aromatic hydrocarbon (PAH) that is formed by incomplete combustion of organic materials. It is found in large amounts in diet, air pollution, cigarette smoke and some occupational atmospheres [1]. Human exposure to this widespread environmental contaminant has been correlated to various pathological situations such as cancer development, and inflammation contributing to cardiovascular and pulmonary diseases [2,3]. Therefore, B[*α*]P has been classified as a priority toxicant by the United States Environmental Protection Agency, the World Health Organization and the European Union. Most of the B[*α*]P-related toxic effects have been linked to the activation of the aryl hydrocarbon receptor (AhR) and its subsequent binding to specific xenobiotic responsive elements within the promoter of responsive genes [4]. For example, the activation of the AhR-related signaling pathway by B[*α*]P results in the up-regulation of xenobiotic metabolizing enzymes such as cytochromes P-450 (CYPs) 1A1, 1A2 and 1B1; this in turn bioactivates B[*α*]P into epoxide derivatives that largely account in a major way for carcinogenic effects in several organs such as lung, liver and lymphoid tissue [5].

Among cell types targeted by B[*α*]P, lymphocytes, in particular T cells which express AhR and possess a CYP1-metabolizing system [6,7] appear to be a major one. Early in vitro and in vivo studies using rodent models reported B[*α*]P-mediated immunotoxic effects such as suppression of antibody production in response to a T-dependent antigen [8], decrease in T-cell responses to mitogens [9], or inhibition of cytotoxic T-lymphocyte generation and natural killer cell activity [10]. Such alterations of immune resistance mechanisms that interfere with T lymphocyte functions have been postulated to facilitate tumor development in responsive animals [11]. In humans, the B[*α*]P capacity to modulate immune function has been less characterized, and data that specifically demonstrate its immunosuppressive effects towards response of human T lymphocytes, are more limited [12,13]. Most studies using human T lymphocytes mainly reported the presence of DNA adducts upon B[*α*]P exposure that may influence cancer development [14,15]. However, the link with T lymphocyte immune response remains to be deciphered. Recently, we have reported an up-regulation of AhR expression and activity in primary cultures of human T lymphocytes by the physiologically relevant T-cell stimulation by anti-CD3 and anti-CD28 antibodies [7]. We have also demonstrated that this up-regulation is associated with an increased capacity to metabolize PAHs such as B[*α*]P, thereby producing specific DNA damage and increasing mutation frequency [16]. Altogether, our data propose primary activated human T lymphocytes as a good model to analyze human health issues depending on the environment. Using a microarray-based transcriptome analysis of activated human T lymphocytes, the present study aims at characterizing global transcriptional alterations after exposure to B[*α*]P, and identifying signaling pathways and biological functions affected by this PAH. We report that B[*α*]P impacts essential functions of adaptive immune responses such as the interferon (IFN) signaling pathway and T lymphocyte recruitment.

## 2. Results

### 2.1. B[α]P Exposure Alters the Expression of 158 Genes in Human T Lymphocytes

To determine the effect of B[*α*]P on the gene signature in human T lymphocytes, primary cultures of T lymphocytes purified from healthy blood donors and activated with anti-CD3 and anti-CD28 antibodies for 72 h, were used and co-exposed to 2 µM B[*α*]P for the last 48 h. We previously reported an early and functional up-regulation of the AhR by such human T lymphocyte activation during 24 h [7], followed by a maximal response of target genes such as *CYP1A1* and *CYP1B1* after exposure to 2 µM B[*α*]P [7,16]. Such a 2 µM concentration is in the range of B[*α*]P concentrations known to lead to maximal response of target genes in cultured human cells [17,18]. In addition and in agreement with our previous results, such a B[*α*]P exposure for 48 h did not significantly alter lymphocyte viability [7,16].

**Figure 1:**
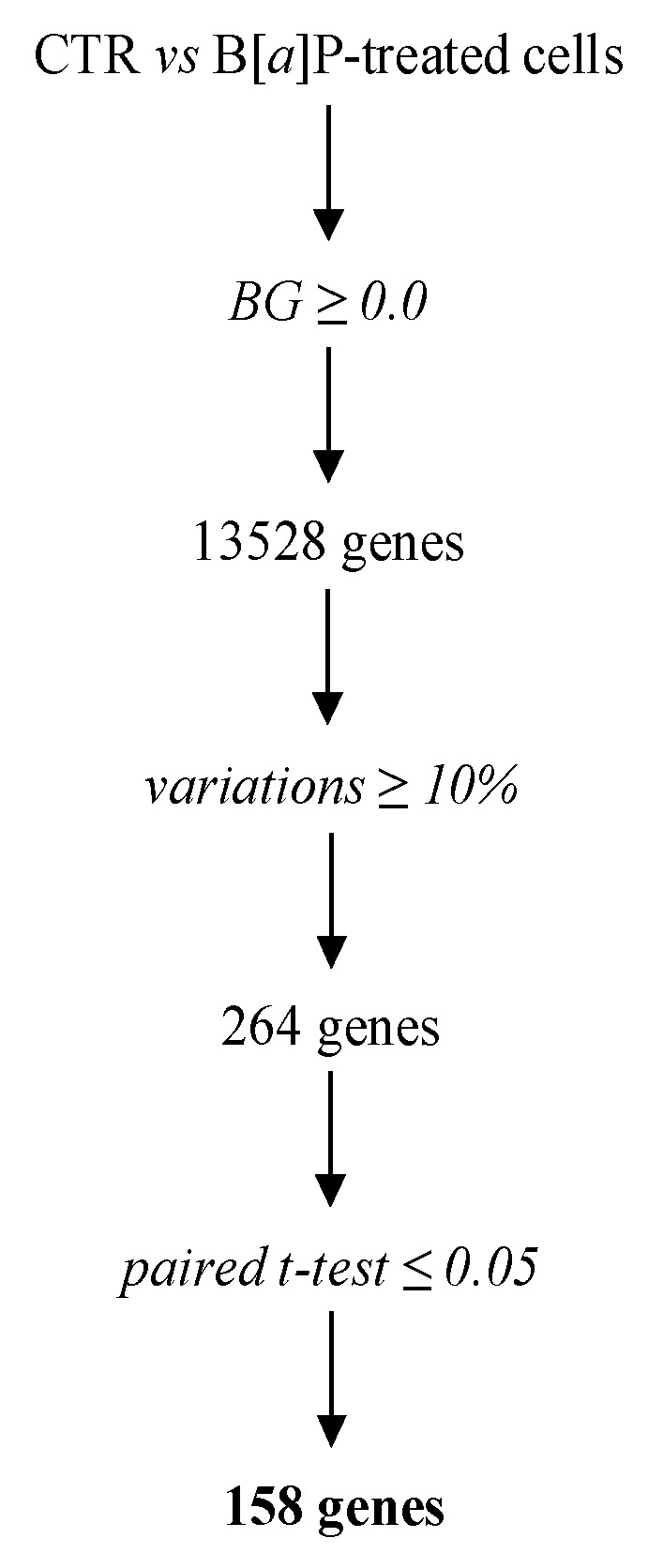
Transcriptomic analysis of differentially expressed genes in B[*α*]P-treated T lymphocytes. A flow chart outlines our procedure to filter genes that display statistically significant signal changes when DMSO (CTR) and B[*α*]P-treated samples were compared.

Using equal amounts of RNAs pooled from 16 independent T lymphocyte cultures into 4 equimolar pools and considering the criteria of selection described in the Materials and Methods section, we were able to identify 158 genes displaying significant signal changes after a 48 h treatment with 2 µM B[*α*]P (Figure 1). The full list of these genes is given in Supplementary Table S1. Among these differentially expressed genes, 97 and 61 were found to be up-regulated and down-regulated upon B[*α*]P treatment, respectively. The top 15 most up-regulated and down-regulated genes are summarized in Table 1. Among these genes, as expected [7,16], *CYP1A1* and *CYP1B1*, well-known targets of AhR [4], appear as the top genes over-expressed after exposure to B[*α*]P, thus validating our experimental conditions.

**Figure 2:**
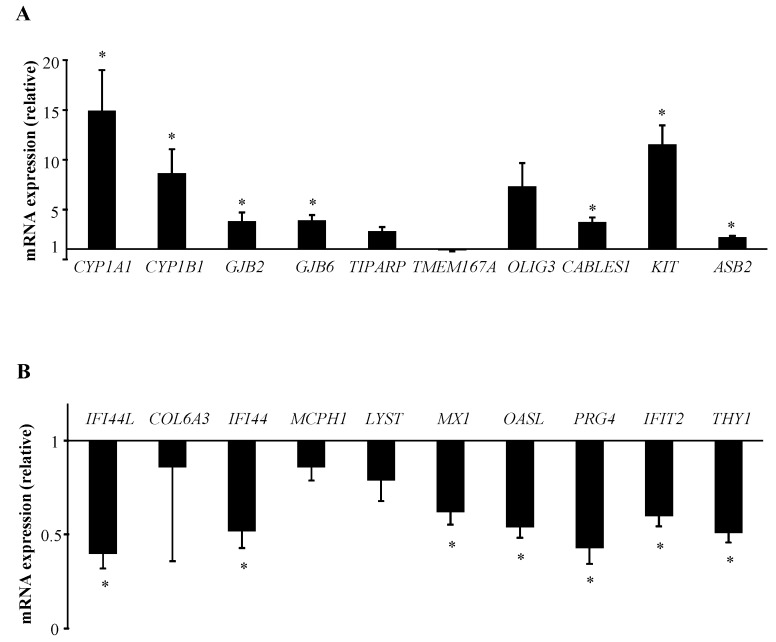
Validation by RT-qPCR analysis of the 10 most significantly up-regulated (**A**) and the 10 most significantly down-regulated (**B**) genes identified by microarray experiments after treatment of human T lymphocytes with 2 µM B[*α*]P for 48 h. Primary human T lymphocytes activated for 72 h were treated with DMSO (CTR) or with 2 µM B[*α*]P for the last 48 h of culture. mRNA expression was analyzed using RT-qPCR. Data are expressed relative to mRNA expression level in CTR T lymphocytes, arbitrarily set at 1 unit, and are shown as mean ± SEM of 4 independent experiments performed on the same pools of RNAs isolated from CTR or B[*α*]P-treated cultures as those used in microarray experiments, with triplicate per experiment. * *p* ≤ 0.05 when compared with CTR T lymphocytes (paired Student’s *t* test).

We then performed RT-qPCR assays using the same equimolar pools of RNAs as those in microarray experiments, in order to confirm transcriptional changes observed for candidate genes selected from the transcriptome analysis. We thus selected the most significantly affected genes: *CYP1A1* and *1B1*, gap junction beta-2 (*GJB2*) and beta-6 (*GJB6*) proteins, TCDD-inducible poly(ADP-ribose) polymerase (*TIPARP*), transmembrane protein 167A (*TMEM167A*), oligodendrocyte transcription factor 3 (*OLIG3*), cyclin-dependent kinase-binding protein (*CABLES1*), proto-oncogene *c-KIT* and E3 ubiquitin ligase specificity subunit (*ASB2*) for the 10 most up-regulated genes; IFN-induced proteins 44L (*IFI44L*), 44 (*IFI44*), p78 (*MX1*) with tetratricopeptide repeats 2 (*IFIT2*), collagen (*COL6A3*), microcephalin *MCPH1*, lysosomal trafficking regulator (*LYST*), enzyme oligoadenylate synthetase like (*OASL*), proteoglycan (*PRG4*), and cell surface antigen (*THY1*) for the 10 most down-regulated ones. The direction of changes in mRNA expression determined by RT-qPCR closely matched the microarray data for all the genes tested, except for *TMEM167A* which rather showed a down-regulated expression (Figure 2). Thus, *CYP1A1*, *CYP1B1*, *GJB2, GJB6*, *TIPARP*, *OLIG3*, *CABLES1*, *KIT* and *ASB2* transcriptional levels were confirmed to be up-regulated after B[*α*]P exposure; with inductions reaching a significant level for all these genes except for *OLIG3* and *TIPARP* (Figure 2A). RT-qPCR analysis also reported the decrease in expression of the most down-regulated genes identified by our microarray study with a significant effect for *IFI44L*, *IFI44*, *MX1*, *OASL*, *PRG4*, *IFIT2* and *THY1* (Figure 2B).

### 2.2. The AhR and IFN Signalling are the Most Significant Canonical Pathways Regulated by B[α]P in Human T Lymphocytes

In order to investigate global signaling pathways affected by B[*α*]P exposure, the 97 up-regulated and the 61 down-regulated transcripts were next submitted to the IPA software. This analysis revealed that “AhR signaling”, “Protein Kinase A signaling”, “Estrogen-mediated S-phase entry” and “Cell cycle: G1/S checkpoint regulation” pathways were the four most significant pathways in the gene set up-regulated by B[*α*]P exposure. Regarding the down-regulated genes, the “IFN signaling”, the “granulocyte adhesion and diapedesis”, the “role of pattern recognition receptors in recognition of bacteria and viruses”, and the “activation of IFN regulatory transcription factor (IRF) by cytosolic pattern recognition receptors’ pathways were the most significant pathways regulated by B[*α*]P exposure (Table 2).

**Figure 3:**
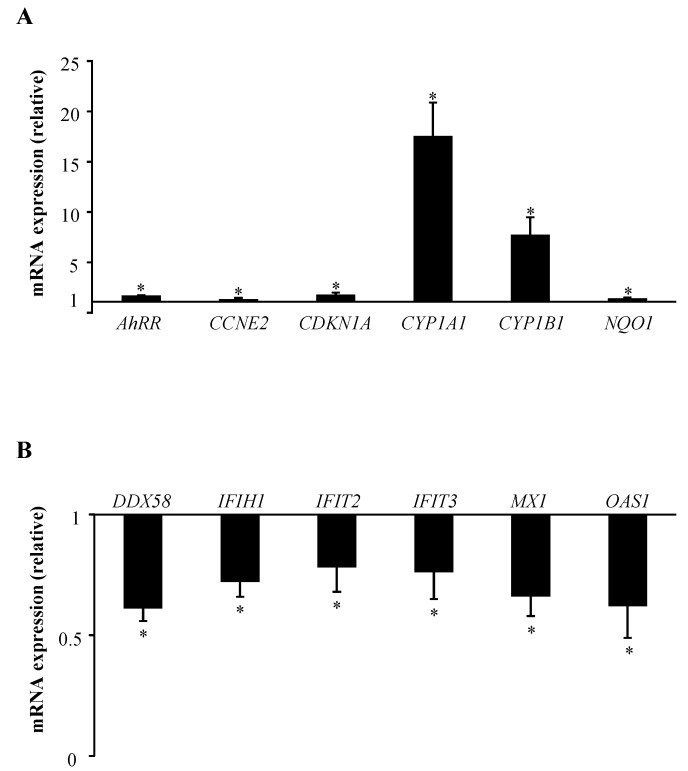
Validation by RT-qPCR analysis of AhR (**A**) and IFN (**B**) signaling-related genes identified by microarray experiments after treatment of human T lymphocytes with 2 µM B[*α*]P for 48 h. Primary human T lymphocytes activated for 72 h were treated with DMSO (CTR) or with 2 µM B[*α*]P for the last 48 h of culture. mRNA expression was analyzed using RT-qPCR. Data are expressed relative to mRNA expression level in CTR T lymphocytes, arbitrarily set at 1 unit, and are shown as mean ± SEM of 16 independent experiments performed on the individual RNAs isolated from CTR or B[*α*]P-treated cultures, with triplicate per experiment. * *p* ≤ 0.05 when compared with CTR T lymphocytes (paired Student’s *t* test).

Among genes belonging to “AhR signaling”, we recently reported the regulation of *CYP1A1*, *CYP1B1* and the cyclin-dependent kinase inhibitor 1A (*CDKN1A*) in human T lymphocytes upon exposure to B[*α*]P [16]. The AhR repressor (*AhRR*), the cyclin E2 (*CCNE2*) and the NAD(P)H dehydrogenase (*NQO1*) have already been reported to be robust targets of B[*α*]P but, in different cell types, but not in primary T lymphocytes [19,20]. We then validated by RT-qPCR assays these changes in *AhRR*, *CCNE2*, *CDKN1A*, *CYP1A1*, *CYP1B1*, and *NQO1* gene expression changes using the 16 individual RNA samples isolated from CTR and B[*α*]P-treated T lymphocyte cultures (Figure 3A). Interestingly, “IFN signaling” and “activation of IRF by cytosolic pattern recognition receptors” pathways were found to be closely associated with genes exhibiting a significant down-regulation in human T lymphocytes treated with B[*α*]P (Table 2). RT-qPCR analysis further validated the gene expression down-regulations of *IFIT3*, *MX1*, *OAS1* expressions (all related to “IFN signaling”) and of the RNA helicase (*DDX58*), the IFN-induced protein with helicase C domain (*IFIH1*) and *IFIT2* (all belonging to “activation of IRF by cytosolic pattern recognition receptors”) (Figure 3B), using the same 16 RNA samples as for AhR signaling-related genes (Figure 3B). Such an analysis identifies these IFN-related proteins as novel target genes for B[*α*]P.

### 2.3. IPA Functional Analysis Revealed the Prominence of Categories Related to Cellular Movement for B[α]P-Regulated Genes in Human T Lymphocytes

With the aim of better understanding the biological relevance of the changes in the expression of 158 genes upon B[*α*]P exposure, IPA software was used to analyze the most significant diseases and disordered biological functions regulated in T lymphocytes after B[*α*]P exposure. As shown in Table 3, the five most significant diseases and disorders revealed by such an analysis were cancer, haematological disease, immunological disease, organismal injury and abnormalities, and antimicrobial response. Cellular movement, cell death and survival, cellular function and maintenance, cell-to-cell signaling and interaction and cellular growth and proliferation were the top five significant molecular and cellular functional categories found to be altered in human T lymphocytes after a 48 h treatment with B[*α*]P (Table 3). Immune cell trafficking, hematological system development and function, tissue morphology, digestive system development and function and lymphoid tissue structure and development were the five most significant categories in physiological development and system function associated with B[*α*]P-modulated genes in T lymphocytes (Table 3). 

**Figure 4:**
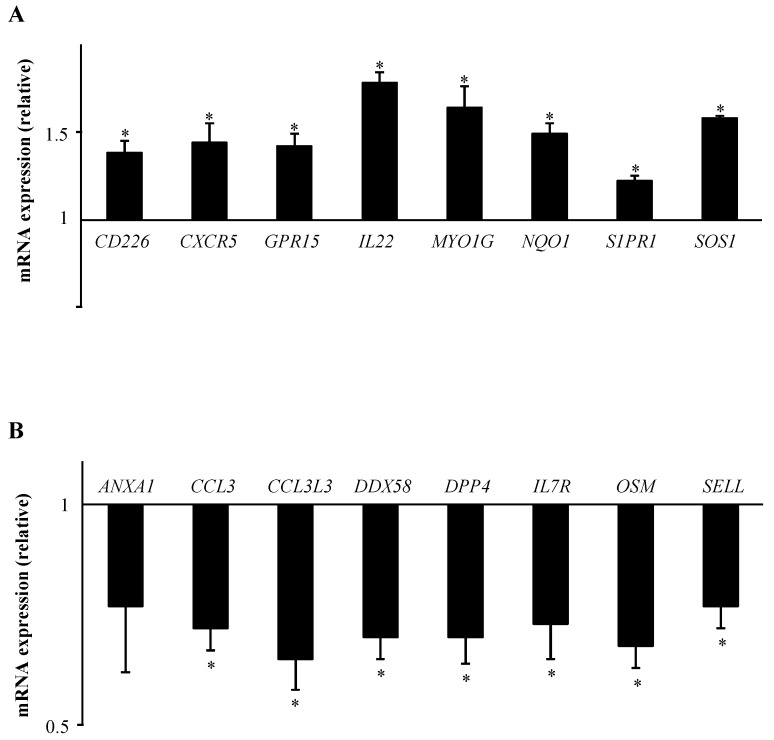
Validation by RT-qPCR analysis of cellular movement-related genes identified by microarray experiments after treatment of human T lymphocytes with 2 µM B[*α*]P for 48 h. Primary human T lymphocytes activated for 72 h were treated with DMSO (CTR) or with 2 µM B[*α*]P for the last 48 h of culture. mRNA expression was analyzed using RT-qPCR. Data are expressed relative to mRNA expression level in CTR T lymphocytes, arbitrarily set at 1 unit, and are shown as mean ± SEM of 4 independent experiments performed on the same pools of RNAs isolated from CTR or B[*a*]P-treated cultures as those used in microarray experiments, with triplicate *per* experiment. * *p* ≤ 0.05 when compared with CTR T lymphocytes (paired Student’s *t* test).

Interestingly, these IPA functional data revealed the prominence of categories related to lymphocyte movement for B[*α*]P-regulated genes. Based on that, we then decided to focus on gene sets linked to this function. Consistent with our transcriptomic data, and using RT-qPCR on the same pools of RNA as those used in microarray experiments, mRNA expressions of the surface receptors *CD226*, *CXCR5*, *GPR15*, and *S1PR1*, the interleukin *IL-22*, the plasma-membrane-associated class I myosin *MYO1G*, *NQO1*, and the membrane-bound guanine nucleotide binding protein *SOS1* were found to be significantly up-regulated after exposure to B[*α*]P (Figure 4A). We also confirmed the decreased mRNA expressions of the annexin A1 (ANXA1), the chemokines (C-C motif) ligands CCL3, CCL3L3, DDX58, the membrane dipeptidyl peptidase 4 (DPP4), the interleukin 7 receptor (IL7R), the oncostatin M (OSM) and the leukocyte-endothelial cell adhesion molecule (SELL), with all of these repressions reaching a significant level, except for ANXA1 (Figure 4B).

### 2.4. B[α]P Inhibits Human T Lymphocyte Chemotaxis and Transendothelial Migration

**Figure 5:**
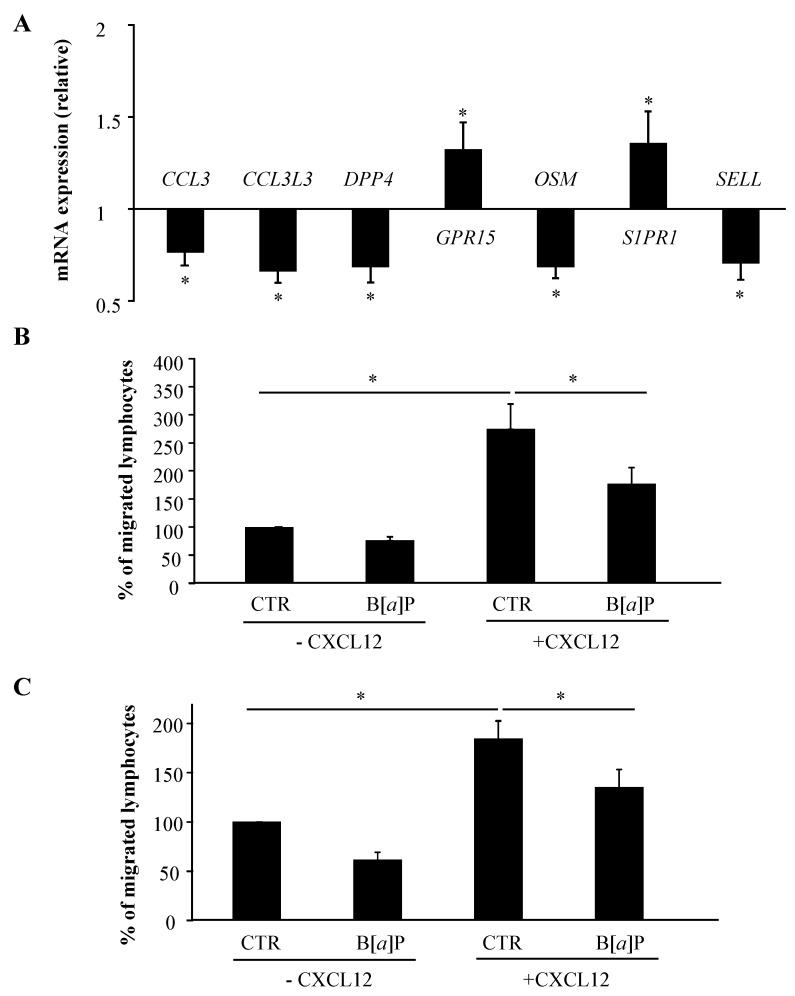
Effect of B[*α*]P on chemotaxis and endothelial transmigration of human T lymphocytes. Primary human T lymphocytes activated for 72 h were treated with DMSO (CTR) or with 2 µM B[*α*]P for the last 48 h of culture. (**A**) mRNA expression was analyzed using RT-qPCR. Data are expressed relative to mRNA expression level in CTR T lymphocytes, arbitrarily set at 1 unit, and are shown as mean ± SEM of 16 independent experiments performed on the individual RNAs isolated from CTR or B[*α*]P-treated cultures, with triplicate per experiment. * *p* ≤ 0.05 when compared with CTR T lymphocytes (paired Student’s *t* test). (**B**, **C**) T lymphocytes were then used for chemotaxis (**B**) and transendothelial migration (**C**) assays with or without CXCL12. Data are expressed as percentages of migrated lymphocytes found in CTR T lymphocytes without CXCL12, and are shown as means ± SEM of 6 independent experiments with duplicate *per* experiment. * *p* ≤ 0.05 when compared with CTR T lymphocytes (analysis of variance followed by Student-Newman-Keuls’s multirange test).

Based on its effects on the mRNA expression of genes that are known to be related to lymphocyte movement, B[*α*]P was thus identified as a potential modulator of T lymphocyte recruitment. To gain insight into this role of B[*α*]P, we first used the 16 individual RNA samples isolated from CTR and B[*α*]P-treated T lymphocytes to validate the unexpected regulation of specific genes linked to T cell homing and trafficking, i.e., CCL3, CCL3L3, DPP4, GPR15, OSM, S1PR1 and SELL. As shown in Figure 5A, mRNA expressions of *GPR15* and *S1PR1* were significantly up-regulated, whereas those of *CCL3*, *CCL3L3*, *DPP4*, *OSM* and *SELL* were down-regulated in human T lymphocytes after a 48-h treatment with B[*α*]P. These genes therefore represent novel target genes for B[*α*]P, and highlight the possible interplay between B[*α*]P exposure and altered T lymphocyte recruitment. This next prompted us to further explore the effect of B[*α*]P on the migration of primary human T lymphocytes by performing functional experiments. The molecular processes involved in T lymphocyte migration are not only chemotaxis, mainly mediated by chemokines/cytokines, but also cellular adhesion, involving selectins or surface receptors [21]. We first developed a transwell assay to investigate the possible regulation of T lymphocytes chemotaxis by B[*α*]P. To do so, we used a gelatin-precoated 8 µm pore cell culture insert in which primary human T lymphocytes, treated or not with B[*α*]P, were allowed to migrate to the basolateral compartment of the transwell system either containing or not containing CXCL12, an highly efficient chemoattractant for T lymphocytes classically used in chemotaxis assays [22]. As shown in Figure 5B, CXCL12 clearly increased the percentage of migrated T lymphocytes, confirming the response of T lymphocytes to this cytokine, and B[*α*]P significantly inhibited this CXCL12-induced chemotaxis. We next developed another transwell assay to evaluate T lymphocyte migration through the blood vessel wall. Therefore, we compared the ability of CTR and B[*α*]P-treated T lymphocytes to migrate across Tumor Necrosis Factor-α-stimulated HMEC-1 in response or not to CXCL12, which also plays an important role in the transendothelial migration of T cells in response to immune processes [23]. Interestingly, B[*α*]P decreased T lymphocyte migration across membrane coated with activated endothelial cells, with a significant inhibition in response to CXCL12 (Figure 5C). Altogether, these data indicate that B[*α*]P cause a significant inhibition of the CXCL12-induced chemotaxis and transendothelial migration of T lymphocytes, therefore identifying a novel immunotoxic effect of this contaminant.

## 3. Discussion

The aim of the present study was to characterize the global transcriptional response of human T lymphocytes to B[*α*]P. To the best of our knowledge, this study provides the first insight into the global transcriptional activity that underlies the effects of B[*α*]P in activated primary human T lymphocytes, a relevant model for human health issues. Indeed, we previously reported that physiological activation of human T lymphocytes by anti-CD3 and anti-CD28 antibodies is of great interest towards the exposure to PAHs, such as B[*α*]P [7,16], thus supporting their genotoxic effects and reinforcing the link suggested between PAH exposure and risk of T lymphoma [24]. However, in this model, the immunotoxic effects of PAHs remain incompletely characterized. Our microarray data indicated that a 48-h treatment of primary human T lymphocytes with B[*α*]P resulted in 158 significantly regulated genes. This represents around 1% of the analyzed genes but we cannot exclude the possibility that other treatment conditions in terms of timing might result in an amplified effect in T lymphocytes. Interestingly, gene expression has been previously reported to be tightly regulated in activated human T lymphocytes through rapid mRNA decay as a part of an homeostatic mechanism to down-modulate an immune response, thus reflecting transient responses in this model [25]. Additionally, except for the AhR target genes, *CYP1A1* and *CYP1B1* [4], few of the 158 differentially expressed transcripts displayed strong amplitude in the changes observed. Compression of level expression in microarray has already been reported [26], and could be due to stringent normalization conditions. Nevertheless, validation by RT-qPCR assays showed that a large number of the 158 regulated transcripts identified by our microarray experiments was confirmed to be differentially expressed in human T lymphocytes after B[*α*]P treatment, therefore demonstrating the global robustness of this transcriptomic analysis.

Among genes identified by our study, some have already been described to be targeted by B[*α*]P. This is notably true for *CYP1A1*, *CYP1B1*, the ferredoxin reductase (*FDXR*), *NQO1*, *CDKN1A*, *AhRR*, *ASB2* or *TIPARP* up-regulated genes [17,18,19,27,28] and *MX1* or the solute carrier *SLC25A37*, regarding the down-regulated ones [29,30]. All these genes therefore appear as robust PAH targets. Many target genes reported in the present work have never been identified as B[*α*]P targets in previous studies investigating its transcriptional signature. Our study allows us to identify novel B[*α*]P target genes such as the connexins *GJB2*, *GJB6*, and the IFN-induced proteins IFI44L, *IFI44*, *IFIH1*, *IFIT2* or *IFIT3*. These identifications likely support the concept of a cell-specific genomic response to PAHs, therefore discarding the idea of a universal battery of AhR-responsive genes as already reported [31]. In further support to that concept, we previously analyzed the transcriptional signature of human macrophages exposed to B[*α*]P [18], and found that the degree of overlap between B[*α*]P-induced expression profiles of macrophages and T lymphocytes was surprisingly small. Nevertheless, AhR- and p53-target genes such as *CYP1A1*, *CYP1B1*, *AhRR*, *ASB2* or *CDKN1A* and the ribonucleotide reductase *RRM2B* were shown to be modulated in both type of cells, all encoding enzymes important in the detoxication and the DNA damage response induced after B[*α*]P exposure [16,18]. 

Beyond the identification of individual genes, our analysis also focused on the identification and characterization of pathways and functions altered after B[*α*]P treatment of human T lymphocytes. AhR signaling is the most significant canonical pathway activated by a 48-h exposure to 2 µM B[*α*]P. This finding is not surprising since B[*α*]P is well-known to act as a potent ligand of AhR [4] and indicate that a notable number of genes induced by B[*α*]P in human T lymphocytes is under the control of AhR. However, since AhR has the ability to act not only as a transcription factor, but also as a signaling mediator [32], the dependence on AhR genomic or non-genomic signaling remains to be elucidated. Regarding the down-regulated genes, prominence of the IFN signaling pathway for enrichment was surprising. IFNs are cytokines exhibiting important roles in the immune response to confer an antiviral state in cells on viral infection [33]. Interestingly, constitutive AhR signaling leading to *TIPARP* up-regulated expression has previously been shown to negatively regulate the type I IFN response during infections with various types of virus in mice [34]. More recently, Guan et al. [35] reported a suppression of IFN-γ production by B[*α*]P in activated mouse T lymphocytes, which contribute to its immunotoxic effects. In the present study, we propose a link between PAH exposure and down-regulation of the IFN response in human T lymphocytes, which might potentially explain the increased incidence of respiratory viral infections after exposure to these environmental air pollutants [36]. In addition, and with respect to the biological functions altered by B[*α*]P, processes related to T lymphocyte recruitment appear as the most affected, leading us to propose B[*α*]P as a modulator of T cell migration. Indeed, the identification, as novel B[*α*]P targets, of chemokines/cytokines such as *CCL3*, *CCL3L3* or *OSM*, and of the surface receptors like *DPP4*, *GPR15*, *S1PR1* and *SELL*, underlines the link previously suggested in the human T-lymphoid cell line Jurkat [37] between B[*α*]P exposure and lymphocyte recruitment. Among these genes, *CCL3*, *CCL3L3*, *DPP4*, *OSM* and *SELL* are down-regulated by B[*α*]P. Interestingly, *CCL3* is a chemokine that not only mediates immune cell chemotaxis, but also regulates T lymphocyte function and migration following viral infection [38]; the cytokine *OSM* has been proposed to control the emigration of lymphocytes through a sustained expression of selectin [39], and the homing receptor *SELL* appears crucial for T lymphocyte activation and migration from lymph nodes, as reported in lymphoma [40]. In agreement with these down-regulated expressions, the data reported in the present study indicate that B[*α*]P significantly inhibited migration of human T lymphocytes towards CXCL12 gradients. This emerging role of B[*α*]P as an inhibitor of T lymphocyte responsiveness to CXCL12 could also be postulated as a novel immunosuppressive effect of B[*α*]P leading to the increased susceptibility to infections upon exposure to environmental air pollutants [36]. However, the molecular mechanism by which B[*α*]P reduces the CXCL12-induced chemotaxis and migration of T lymphocytes, remains to be elucidated. As classically reported for B[*α*]P-induced immunosuppressive effects [12,18], AhR and CYP1-dependent metabolism might play a major role in these inhibitory effects. Nevertheless, a down-regulated expression of proteins involved in T lymphocyte motility by B[*α*]P [37], as well as an inhibition of migration in response to CXCL12 by another AhR ligand, the flavonoid genistein [41], have been reported in Jurkat cells showing no detectable AhR and B[*α*]P metabolites [42], thus suggesting that AhR activation and mechanisms-unrelated to B[*α*]P metabolism may account for these inhibitory effects towards migration. In addition, since IFN has been shown to play a critical role in T lymphocyte recruitment [43,44], it will also be interesting to further explore the role of IFN signaling in this decreased T lymphocyte migration.

Besides the interest in better understanding molecular bases of PAH toxicity, the identification of new B[*α*]P-target genes in lymphocytes also provides potential new biomarkers of exposure to PAHs, which may be convenient for biomonitoring exposed subjects. In addition, it should be kept in mind that most of the microarray-based transcriptomic analysis of gene expression changes due to PAHs have been performed using human transformed cell lines or rodent cells [17,27,45]. This therefore opens the question of the relevance of the data obtained in those studies to human. Data obtained with primary human lymphocytes, which are normal non-transformed human cells, may be consequently more adequate to better characterize the response of normal human cells/tissues to environmental contaminants such as B[*α*]P. More detailed studies are needed to clarify the potential of these genes as new biomarkers of exposure to PAHs.

Together these results identified lymphocytic genes whose expression was targeted by B[*α*]P. Analysis of these data indicated that AhR and IFN signaling cascades constitute major canonical pathways activated by B[*α*]P, whereas biological functions linked to T lymphocyte recruitment are among the most affected, thus reinforcing the immunotoxic effects of PAHs.

## 4. Materials and Methods

### 4.1. Cell Culture and Treatment

Peripheral blood mononuclear cells were isolated from blood donor buffy coats (written consent for the use of blood samples for the research protocol obtained according to the regulation for blood transfusion of the French blood organization Etablissement Français du Sang, Rennes (France)) by Ficoll (Thermofischer Scientific, Braunschweig, Germany) gradient centrifugation. After separation of monocytes by a 1-h adhesion step, T lymphocytes were purified from nonadherent cells by negative selection using Dynabeads^®^ Untouched™ Human T Cells Kit (Thermofischer Scientific). T lymphocytes were cultured in RPMI medium (Eurobio, Les Ulis, France) supplemented with 20 IU/mL penicillin, 20 μg/mL streptomycin, and 10% decomplemented fetal calf serum (Thermofischer Scientific), and stimulated with Dynabeads^®^ T-Expander beads coated with anti-CD3 and anti-CD28 antibodies (Thermofischer Scientific) before a 48-h treatment with 2 µM B[*α*]P (Sigma-Aldrich, St. Louis, MO, USA) as previously reported [16]. B[*α*]P was used as stock solutions in dimethylsulfoxide (DMSO). The final concentration of DMSO in culture medium was always <0.2% *v*/*v* and control cultures received a vehicle containing the same concentration of DMSO (CTR) as treated cultures.

### 4.2. Microarray Experiments

#### 4.2.1. RNA Extraction

Total RNA was isolated from T lymphocytes using the TRIzol method (Thermofischer Scientific) and then purified using a RNeasy Mini Kit with on-column RNAse-free DNAse digestion (Qiagen, Courtaboeuf, France), according to the manufacturer’s protocol. RNA was next quantified with the nanodrop ND-1000 spectrophotometer (Nano-Drop Technologies, Rockland, DE, USA), and RNA integrity was assessed with RNA 6000 Nano LabChip kit using the Agilent 2100 Bioanalyzer (Agilent Technologies, Palo Alto, CA, USA). Only RNA with an RNA integrity number >9 was used for further analysis (2100 expert software, Agilent Technologies). Independent T lymphocyte cultures isolated from 16 blood donors, each available in CTR and B[*α*]P-treated conditions were performed. Equal amounts of RNAs were pooled from these cultures to constitute 4 equimolar pools with 4 different blood donors per pool. Pooling small samples for array analysis is considered advantageous in situations where the level of biological variation could be high compared to technical variation on the array [18,46].

#### 4.2.2. Microarray Hybridization

Total RNA was amplified and labelled using the Gene Chip^TM^ WT PLUS Reagent Kit according to the manufacturer’s instructions (ThermoFischer Scientific). For each sample, RNA pools were hybridized to Human Clariom^TM^ S GeneChip (ThermoFischer Scientific). Arrays were immediately scanned, and images were analyzed and rigorously quality controlled for hybridization artefacts.

#### 4.2.3. Data Normalization

The resulting CEL files were processed using the oligo package available performed in R/Bioconductor [47]. Data were then normalized and background corrected using the SCAN.UPC package [48] with the Brainarray custom CDF file for directly mapping Affymetrix probe to Entrez gene identifiers (hta20_Hs_ENTREZG version 21.0.0) [49]. Data were uploaded to the NCBI Gene Expression Omnibus (GEO) repository under the accession number GSE117527 [50]. Lists of genes significantly induced or repressed after B[*α*]P exposure were also uploaded to the TOXsIgN repository under the accession TSP758 [51].

### 4.3. Statistical Filtration of Differentially Expressed Genes

The statistical filtration of the genes differentially expressed between CTR and B[*α*]P-treated samples was performed using the Annotation, Mapping, Expression and Network suite of tools [52]. Briefly, we first filtered genes with at least one signal above the background expression cutoff (≥0.0) and with a minimal variation of 10% between both experimental conditions as determined by the inflection point of the fold-change curve (Supplementary Figure S1). Finally, a paired Student’s *t* test (*p* ≤ 0.05) was used to identify significantly differentially expressed genes which were further classified into two expression patterns: Up-regulated and down-regulated genes after B[*α*]P exposure.

### 4.4. Functional Analysis by Ingenuity Pathway Analysis (IPA)

Lists of genes significantly induced or repressed after B[*α*]P exposure were uploaded into IPA software (IPA, Ingenuity Systems, QIAGEN, available online: www.ingenuity.com) for signaling pathways and biological functions analysis by comparison with the Ingenuity Knowledge Database.

### 4.5. RT-qPCR Assays

Total RNA was isolated from the same independent T lymphocyte cultures isolated from 16 blood donors as used for microarray experiments using the TRIzol method (Thermofischer Scientific) and then reverse-transcribed into cDNA using the RT Applied Biosystems kit (Foster City, CA, USA). qPCR assays were performed using gene-specific primers from Eurogentec (Seraing, Belgium). The amplification curves of the PCR products were analyzed with the ABI Prism SDS software using the comparative cycle threshold method. Relative quantification of the steady-state target mRNA levels was calculated after normalization of the total amount of cDNA tested to a 18S mRNA endogenous reference as previously described [16].

### 4.6. Chemotaxis and Transendothelial Migration Assays

Chemotaxis assays were carried out using 24-well transwell chambers exhibiting 8 µm pores (Dutscher, Brumath, France). Into each transwell pre-coated with 0.1% gelatin, 5 × 10^5^ T lymphocytes in 200 µL were transferred. The transwell was then inserted into a 24-well plate containing a 0.6 mL volume of the chemotaxis medium (RPMI, 1% bovine serum albumine, 2% fetal calf serum) with or without the chemokine (C-X-C motif) ligand 12 (CXCL12) (50 ng/mL) (Preprotech, Rocky Hill, USA). Migration was allowed for 3 h at 37 °C. Lymphocytes which migrated across the membrane were harvested and subsequently counted by Malassez counting slide. For each experiment migration, data have been normalized considering the number of counted cells in CTR conditions as 100%.

For transmigration assays through an endothelial monolayer, 200,000 human immortalized microvascular endothelial cells (HMEC-1)/cm^2^ were seeded onto a 0.1% gelatin pre-coated transwell and allowed to form a complete monolayer on the membrane of 8 µm pore size 24-well transwell in MCDB-131 medium containing epidermal growth factor (10 ng/mL), hydrocortisone (1 µg/mL), glutamine (10 mM) (Thermofischer Scientific), penicillin (50 units/mL) and streptomycin (50 µg/mL) and supplemented with 10% fetal calf serum as previously reported [53]. HMEC-1 barrier was controlled by measuring transendothelial electrical resistance using the Millicell ERS-2^®^ (Merck Millipore, Darmstadt, Germany). HMEC-1 were then activated using Tumor Necrosis Factor-α (Thermofischer Scientific) (10 ng/mL) for 8 h. Into each transwell, 5 × 10^5^ T lymphocytes in 200 µL were transferred and the transwell was inserted into a 24-well plate containing a chemotaxis medium with or without CXCL12 as previously reported for chemotaxis assays. Migration was allowed for 3 h at 37 °C and the number of migrated lymphocytes was evaluated, as described above.

### 4.7. Statistical Analysis

Data are expressed as means ± SEM. Statistical significance of the differences was assessed using GraphPad Instat (GraphPad software, INC., La Jolla, CA, USA) by paired Student’s *t* test or one-way analysis of variance followed by Student-Newman-Keuls post hoc tests. The criterion of significance was *p* ≤ 0.05.

## Figures and Tables

**Table 1:** Top 15 up-regulated and down-regulated genes after treatment of human T lymphocytes with 2 µM B[*α*]P for 48 h.

ID	Gene Name	Description	Differential Expression (log-2) ^a^	*p*-Value
**Top 15 up-regulated genes**				
1543	*CYP1A1*	cytochrome P450, family 1, subfamily A, polypeptide 1	1.152	5.9 × 10^−3^
1545	*CYP1B1*	cytochrome P450, family 1, subfamily B, polypeptide 1	1.058	4.7 × 10^−3^
2706	*GJB2*	gap junction protein, beta 2, 26 kDa	0.702	5.7 × 10^−3^
10804	*GJB6*	gap junction protein, beta 6, 30 kDa	0.528	7.7 × 10^−3^
25976	*TIPARP*	TCDD-inducible poly (ADP-ribose) polymerase	0.528	1.0 × 10^−2^
153339	*TMEM167A*	transmembrane protein 167A	0.491	3.0 × 10^−2^
167826	*OLIG3*	oligodendrocyte transcription factor 3	0.484	2.7 × 10^−2^
91768	*CABLES1*	Cdk5 and Abl enzyme substrate 1	0.426	3.1 × 10^−4^
3815	*KIT*	proto-oncogene c-Kit	0.401	1.2 × 10^−5^
51676	*ASB2*	ankyrin repeat and SOCS box containing 2	0.388	5.6 × 10^−4^
83888	*FGFBP2*	fibroblast growth factor binding protein 2	0.362	2.0 × 10^-3^
5774	*PTPN3*	protein tyrosine phosphatase, non-receptor type 3	0.349	1.1 × 10^−2^
9289	*ADGRG1*	adhesion G protein-coupled receptor G1	0.346	5.8 × 10^-3^
23682	*RAB38*	RAB38, member RAS oncogene family	0.328	4.6 × 10^−3^
81618	*ITM2C*	integral membrane protein 2C	0.307	1.5 × 10^−2^
**Top 15 down-regulated genes**				
10964	*IFI44L*	interferon-induced protein 44-like	−0.451	1.7 × 10^−2^
1293	*COL6A3*	collagen, type VI, alpha 3	−0.337	6.3 × 10^−3^
10561	*IFI44*	interferon-induced protein 44	−0.322	3.2 × 10^−2^
79648	*MCPH1*	microcephalin 1	−0.319	1.0 × 10^−2^
1130	*LYST*	lysosomal trafficking regulator	−0.282	2.2 × 10^−2^
4599	*MX1*	MX dynamin-like GTPase 1	−0.282	4.8 × 10^−2^
8638	*OASL*	2′-5′-oligoadenylate synthetase-	−0.269	2.0 × 10^−3^
3433	*IFIT2*	interferon-induced protein with tetratricopeptide repeats 2	−0.267	5.6 × 10^−4^
10216	*PRG4*	proteoglycan 4	−0.266	3.4 × 10^−2^
7070	*THY1*	Thy-1 cell surface antigen	−0.265	1.0 × 10^−2^
6402	*SELL*	selectin L	−0.233	3.1 × 10^−2^
56479	*KCNQ5*	potassium channel, voltage gated KQT-like subfamily Q, member 5	−0.232	1.7 × 10^−2^
1803	*DPP4*	dipeptidyl-peptidase 4	−0.222	2.3 × 10^−2^
3394	*IRF8*	interferon regulatory factor 8	−0.220	1.2 × 10^−2^
5167	*ENPP1*	ectonucleotide pyrophosphatase/phosphodiesterase 1	−0.207	2.1 × 10^−2^

^a^ Differential expression (log-2) corresponds to the difference in mRNA expression measured in log-2 intensities between B[*α*]P and DMSO (CTR) conditions; they are the medians of 4 microarray experiments, performed on pools of RNAs isolated from CTR or B[*α*]P-treated primary cultures of T lymphocytes established from 16 blood donors (4 different blood donors per pool).

**Table 2:** Top canonical pathways regulated after treatment of human T lymphocytes with 2 µM B[*α*]P for 48 h.

Top Pathways	^a^*p*-Value	^b^ Regulated Genes
**Up-regulated genes**		
Aryl Hydrocarbon Receptor Signaling	3.99 × 10^−5^	*AhRR*, *CCNE2*, *CDKN1A*, *CYP1A1*, *CYP1B1*, *NQO1*
Protein Kinase A Signaling	7.36 × 10^−5^	*ADCY9*, *ADD2*, *DUSP4*, *MYH10*, *MYL9*, *PPP1R14C*, *PTPDC1*, *PTPN3*, *SAMD3*
Estrogen-mediated S-phase entry	2.06 × 10^−4^	*CCNE2*, *CDKN1A*, *E2F7*
Cell cycle: G1/S checkpoint regulation	2.14 × 10^−4^	*CCNE2*, *CDKN1A*, *E2F7*, *SAMD3*
**Down-regulated genes**		
Interferon Signaling	1.41 × 10^−4^	*IFIT3*, *MX1*, *OAS1*
Granulocyte Adhesion and Diapedesis	1.74 × 10^−4^	*CCL3*, *CCL3L3*, *CCL4L1*, *CCL4L2, SELL*, *THY1*
Role of Pattern Recognition Receptors in Recognition of Bacteria and Viruses	5.97 × 10^−4^	*DDX58*, *IFIH1*, *OAS1*, *OSM*
Activation of IRF by Cytosolic Pattern Recognition Receptors	7.42 × 10^−4^	*DDX58*, *IFIH1*, *IFIT2*

^a^*p*-values were calculated using the IPA software. ^b^ The list of genes whose expression was altered by B[*α*]P treatment was given for each pathway by the IPA software.

**Table 3:** Top diseases and biological functions regulated after treatment of human T lymphocytes with 2 µM B[*α*]P for 48 h.

Network	Top Functions	*p*-Value ^a^	Focus Genes ^b^
**Diseases and Disorders**			
1	Cancer	1.82 × 10^−3^–1.61 × 10^−12^	144
2	Hematological Disease	1.64 × 10^−3^–1.61 × 10^−12^	67
3	Immunological Disease	1.64 × 10^−3^–1.61 × 10^−12^	73
4	Organismal Injury and Abnormalities	1.82 × 10^−3^–1.61 × 10^−12^	147
5	Antimicrobial Response	2.89 × 10^−4^–2.23 × 10^−10^	19
**Molecular and Cellular functions**			
1	Cellular Movement	1.77 × 10^−3^–6.81 × 10^−12^	60
2	Cell Death and Survival	1.59 × 10^−3^–2.05 × 10^−11^	77
3	Cellular Function and Maintenance	1.30 × 10^−3^–7.97 × 10^−11^	52
4	Cell-To-Cell Signaling and Interaction	1.82 × 10^−3^–4.36 × 10^−8^	41
5	Cellular Growth and Proliferation	1.68 × 10^−3^–5.20 × 10^−8^	77
**Physiological system development and function**			
1	Immune Cell Trafficking	1.82 × 10^−3^–6.81 × 10^−12^	45
2	Hematological System Development and Function	1.82 × 10^−3^–5.20 × 10^−10^	64
3	Tissue Morphology	1.67 × 10^−3^–2.55 × 10^−9^	56
4	Digestive System Development and Function	1.07 × 10^−3^–1.75 × 10^−7^	25
5	Lymphoid Tissue Structure and Development	1.78 × 10^−3^–1.75 × 10^−7^	48

^a^*p*-values were calculated using the IPA software. ^b^ The number of genes whose expression was altered by B[*α*]P treatment was given for each diseases and functions by the IPA software.

## Supplementary Materials

Supplementary materials can be found at http://www.mdpi.com/1422-0067/19/11/3626/s1. Table S1: Genes differentially regulated after treatment of human T lymphocytes with 2 μM B[*α*]P for 48 h. Figure S1: Selection criteria for microarray data.
