# Supplementary material for: Genome-Wide Transcriptional and Functional Analysis of Human T Lymphocytes Treated with Benzo[α]pyrene
Source: Int J Mol Sci. 2018 Nov 17;19(11):3626. doi: 10.3390/ijms19113626 (PMC6274903; doi:10.3390/ijms19113626)
Supplement: Supplementary file 1 [file ijms-19-03626-s001.zip › Suppl. Fig.1.pdf]

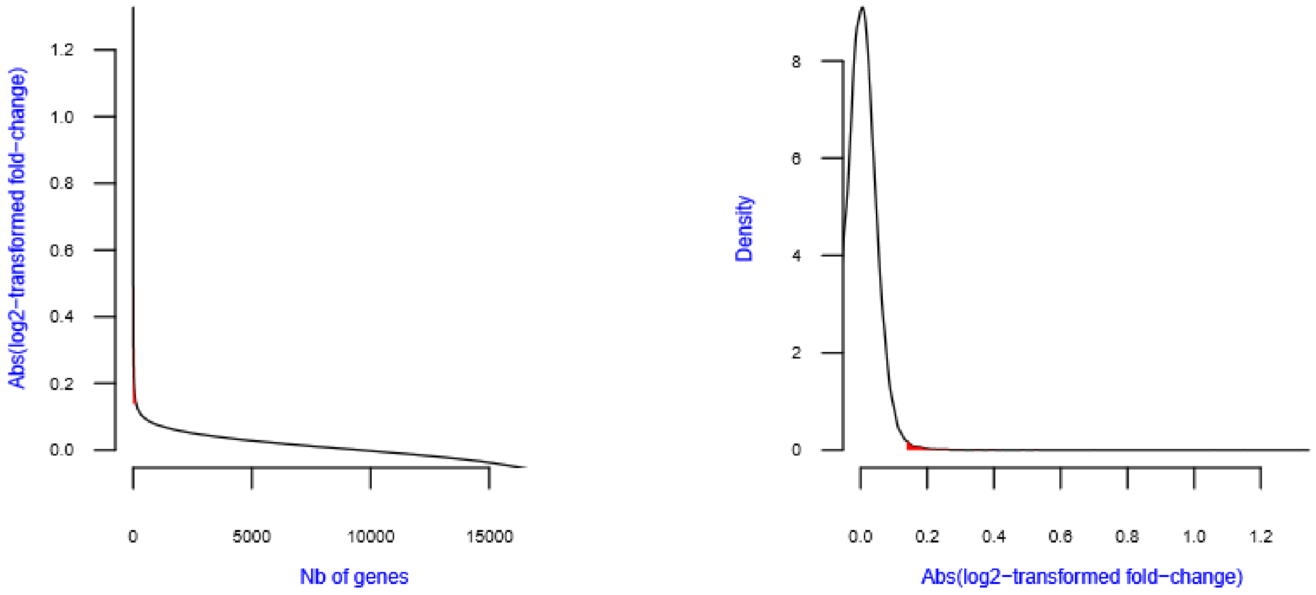

Supplementary Figure S1: Selection criteria for microarray data. The filtered genes with at least one signal above the background expression cutoff ($\geq$ 0.0) and with a minimal variation of 10% between both experimental conditions were determined by the inflection point of the fold-change curve.
