# Supplementary material for: Genome-Wide Transcriptional and Functional Analysis of Human T Lymphocytes Treated with Benzo[α]pyrene
Source: Int J Mol Sci. 2018 Nov 17;19(11):3626. doi: 10.3390/ijms19113626 (PMC6274903; doi:10.3390/ijms19113626)
Supplement: Supplementary file 1 [file ijms-19-03626-s001.zip › Suppl. Table 1.pdf]

Supplemental Table S1 : Genes differentially regulated after treatment of human T lymphocytes with 2 µM B[*a*]P for 48 h

ID	GeneName	Description	Differential expression (log-2)
23461	*ABCA5*	ATP-binding cassette, sub-family A (ABC1), member 5;ATP-binding cassette A5	0,142
10257	*ABCC4*	ATP-binding cassette, sub-family C (CFTR/MRP), member 4;MRP/cMOAT-related ABC transporter;bA464I2.1 (ATP-binding cassette, sub-family C (CFTR/MRP), member 4);canalicular multispecific organic anion transporter (ABC superfamily);multi-specific organic anion transporter B	-0,141
2180	*ACSL1*	acyl-CoA synthetase long-chain family member 1;LACS 1;LACS 2;acyl-CoA synthetase 1;fatty-acid-Coenzyme A ligase, long-chain 1;fatty-acid-Coenzyme A ligase, long-chain 2;lignoceroyl-CoA synthase;long-chain acyl-CoA synthetase 1;long-chain acyl-CoA synthetase 2;long-chain fatty acid-CoA ligase 2;long-chain fatty-acid-coenzyme A ligase 1;palmitoyl-CoA ligase 1;palmitoyl-CoA ligase 2;paltimoyl-CoA ligase 1	-0,160
115	*ADCY9*	adenylate cyclase 9;ATP pyrophosphate-lyase 9;adenylate cyclase type IX;adenylyl cyclase 9;type IX ATP pyrophosphate-lyase	0,178
119	*ADD2*	adducin 2 (beta);erythrocyte adducin subunit beta	0,155
976	*ADGRE5*	adhesion G protein-coupled receptor E5;CD97 molecule;leukocyte antigen CD97;seven transmembrane helix receptor;seven-span transmembrane protein;seven-transmembrane, heterodimeric receptor associated with inflammation	-0,138
9289	*ADGRG1*	adhesion G protein-coupled receptor G1;7-transmembrane protein with no EGF-like N-terminal domains-1;G protein-coupled receptor 56	0,346
133	*ADM*	adrenomedullin;preproadrenomedullin;proadrenomedullin N-20 terminal peptide	0,196
3899	*AFF3*	AF4/FMR2 family, member 3;MLLT2-related protein;lymphoid nuclear protein 4;lymphoid nuclear protein related to AF4;protein LAF-4	-0,140
57491	*AHRR*	aryl-hydrocarbon receptor repressor;ahR repressor;aryl hydrocarbon hydroxylase regulator;class E basic helix-loop-helix protein 77;dioxin receptor repressor	0,156
301	*ANXA1*	annexin A1;annexin I (lipocortin I);annexin-1;calpactin II;calpactin-2;chromobindin-9;phospholipase A2 inhibitory protein	-0,158
10123	*ARL4C*	ADP-ribosylation factor-like 4C;ADP ribosylation factor-like protein 7;ADP-ribosylation factor-like 7;ADP-ribosylation factor-like protein 7;ADP-ribosylation factor-like protein LAK	0,186
51676	*ASB2*	ankyrin repeat and SOCS box containing 2;ankyrin repeat and SOCS box-containing protein 2a	0,388
55252	*ASXL2*	additional sex combs like transcriptional regulator 2;additional sex combs like 2;additional sex combs-like protein 2;polycomb group protein ASXH2	-0,177
80162	*ATHL1*	ATH1, acid trehalase-like 1 (yeast)	0,161
55509	*BATF3*	basic leucine zipper transcription factor, ATF-like 3;21 kDa small nuclear factor isolated from T-cells;21-kD small nuclear factor isolated from T cells;B-ATF-3;Jun dimerization protein 1;Jun dimerization protein p21SNFT	-0,158
353500	*BMP8A*	bone morphogenetic protein 8a;BMP-8A	-0,204
664	*BNIP3*	BCL2/adenovirus E1B 19kDa interacting protein 3	0,166
53838	*C11orf24*	chromosome 11 open reading frame 24	0,144
128346	*C1orf162*	chromosome 1 open reading frame 162	-0,160
203228	*C9orf72*	chromosome 9 open reading frame 72	-0,141

91768	*CABLES1*	Cdk5 and Abl enzyme substrate 1;interactor with CDK3 1	0,426
800	*CALD1*	caldesmon 1	0,184
6348	*CCL3*	chemokine (C-C motif) ligand 3;G0/G1 switch regulatory protein 19-1;PAT 464.1;SIS-beta;macrophage inflammatory protein 1-alpha;small inducible cytokine A3 (homologous to mouse Mip-1a);tonsillar lymphocyte LD78 alpha protein	-0,177
414062	*CCL3L3*	chemokine (C-C motif) ligand 3-like 3;G0/G1 switch regulatory protein 19-2;LD78-beta(1-70);chemokine (C-C motif) ligand 3-like, centromeric;small inducible cytokine A3-like 1;tonsillar lymphocyte LD78 beta protein	-0,145
9560	*CCL4L2*	chemokine (C-C motif) ligand 4-like 2;MIP-1-beta;macrophage inflammatory protein 1-beta;macrophage inflammatory protein-1b2;monocyte adherence-induced protein 5-alpha;small inducible cytokine A4-like	-0,203
83605	*CCM2*	cerebral cavernous malformation 2;malcavernin	0,169
9134	*CCNE2*	cyclin E2	0,169
901	*CCNG2*	cyclin G2	0,176
135228	*CD109*	CD109 molecule;150 kDa TGF-beta-1-binding protein;C3 and PZP-like alpha-2-macroglobulin domain-containing protein 7;Gov platelet alloantigens;activated T-cell marker CD109;platelet-specific Gov antigen	0,262
4345	*CD200*	CD200 molecule;CD200 antigen;MRC OX-2 antigen;antigen identified by monoclonal antibody MRC OX-2	-0,165
10666	*CD226*	CD226 molecule;DNAX accessory molecule 1;DNAX accessory molecule-1;T lineage-specific activation antigen 1 antigen;adhesion glycoprotein;platelet and T cell activation antigen 1	0,220
928	*CD9*	CD9 molecule;5H9 antigen;BA-2/p24 antigen;CD9 antigen (p24);cell growth-inhibiting gene 2 protein;leukocyte antigen MIC3;motility related protein-1;tetraspanin-29	0,212
1026	*CDKN1A*	cyclin-dependent kinase inhibitor 1A (p21, Cip1);CDK-interacting protein 1;CDK-interaction protein 1;DNA synthesis inhibitor;melanoma differentiation associated protein 6;wild-type p53-activated fragment 1	0,267
148523	*CIART*	circadian associated repressor of transcription;chIP-derived repressor of network oscillator;computationally highlighted repressor of the network oscillator	0,172
134147	*CMBL*	carboxymethylenebutenolidase homolog (Pseudomonas);carboxymethylenebutenolidase-like (Pseudomonas)	0,152
1293	*COL6A3*	collagen, type VI, alpha 3;collagen VI, alpha-3 polypeptide	-0,337
643	*CXCR5*	chemokine (C-X-C motif) receptor 5;Burkitt lymphoma receptor 1, GTP binding protein (chemokine (C-X-C motif) receptor 5);Burkitt lymphoma receptor 1, GTP-binding protein;CXC-R5;CXCR-5;MDR-15;monocyte-derived receptor 15	0,156
1543	*CYP1A1*	cytochrome P450, family 1, subfamily A, polypeptide 1;CYPIA1;aryl hydrocarbon hydroxylase;cytochrome P1-450, dioxin-inducible;cytochrome P450 form 6;cytochrome P450, subfamily I (aromatic compound-inducible), polypeptide 1;cytochrome P450-C;cytochrome P450-P1;flavoprotein-linked monooxygenase;xenobiotic monooxygenase	1,152
1545	*CYP1B1*	cytochrome P450, family 1, subfamily B, polypeptide 1;aryl hydrocarbon hydroxylase;cytochrome P450, subfamily I (dioxin-inducible), polypeptide 1 (glaucoma 3, primary infantile);flavoprotein-linked monooxygenase;microsomal monooxygenase;xenobiotic monooxygenase	1,058
23586	*DDX58*	DEAD (Asp-Glu-Ala-Asp) box polypeptide 58;DEAD box protein 58;DEAD/H (Asp-Glu-Ala-Asp/His) box polypeptide;RNA helicase RIG-I;retinoic acid-inducible gene 1 protein;retinoic acid-inducible gene I protein	-0,148
91319	*DERL3*	derlin 3;DERtrin 3;DERtrin-3;Der1-like domain family, member 3;degradation in endoplasmic reticulum protein 3;der1-like protein 3	0,168
10202	*DHRS2*	dehydrogenase/reductase (SDR family) member 2;dehydrogenase/reductase member 2;dicarbonyl reductase HEP27;protein D;short chain dehydrogenase/reductase family 25C member 1;short chain dehydrogenase/reductase family 25C, member 1;short-chain alcohol dehydrogenase family member	0,189
10170	*DHRS9*	dehydrogenase/reductase (SDR family) member 9;3-alpha hydroxysteroid dehydrogenase;NADP-dependent retinol dehydrogenase/reductase;retinol dehydrogenase 15;retinol dehydrogenase homolog;short chain dehydrogenase/reductase family 9C member 4;short chain dehydrogenase/reductase family 9C, member 4;short-chain dehydrogenase/reductase retSDR8;tracheobronchial epithelial cell-specific retinol dehydrogenase	-0,150
30836	*DNTTIP2*	deoxynucleotidyltransferase, terminal, interacting protein 2;LPTS-interacting protein 2;acidic 82 kDa protein mRNA;estrogen receptor binding protein;tdT-interacting factor 2	0,139

1803	*DPP4*	dipeptidyl-peptidase 4;ADCP-2;DPP IV;T-cell activation antigen CD26;adenosine deaminase complexing protein 2;dipeptidyl peptidase IV;dipeptidylpeptidase 4;dipeptidylpeptidase IV (CD26, adenosine deaminase complexing protein 2)	-0,222
1846	*DUSP4*	dual specificity phosphatase 4;MAP kinase phosphatase 2;VH1 homologous phosphatase 2;dual specificity protein phosphatase hVH2;mitogen-activated protein kinase phosphatase 2;serine/threonine specific protein phosphatase	0,140
144455	*E2F7*	E2F transcription factor 7;E2F-7	0,181
60401	*EDA2R*	ectodysplasin A2 receptor;EDA-A2 receptor;X-linked ectodysplasin-A2 receptor;tumor necrosis factor receptor superfamily member XEDAR	0,154
5167	*ENPP1*	ectonucleotide pyrophosphatase/phosphodiesterase 1;E-NPP 1;Ly-41 antigen;alkaline phosphodiesterase 1;membrane component chromosome 6 surface marker 1;membrane component, chromosome 6, surface marker 1;phosphodiesterase I/nucleotide pyrophosphatase 1;plasma-cell membrane glycoprotein 1;plasma-cell membrane glycoprotein PC-1	-0,207
2034	*EPAS1*	endothelial PAS domain protein 1;EPAS-1;HIF-1-alpha-like factor;HIF-1alpha-like factor;HIF-2-alpha;HIF2-alpha;PAS domain-containing protein 2;basic-helix-loop-helix-PAS protein MOP2;class E basic helix-loop-helix protein 73;hypoxia-inducible factor 2 alpha;hypoxia-inducible factor 2-alpha;member of PAS protein 2	-0,174
2109	*ETFB*	electron-transfer-flavoprotein, beta polypeptide;beta-ETF;electron transfer flavoprotein beta subunit;electron transfer flavoprotein beta-subunit;electron transfer flavoprotein, beta polypeptide;electron-transferring-flavoprotein, beta polypeptide	0,153
59271	*EVA1C*	eva-1 homolog C (C. elegans);family with sequence similarity 176, member C;protein FAM176C	-0,182
51466	*EVL*	Enah/Vasp-like;ena/vasodilator-stimulated phosphoprotein-like	0,154
2184	*FAH*	fumarylacetoacetate hydrolase (fumarylacetoacetase);FAA;beta-diketonase	0,183
642273	*FAM110C*	family with sequence similarity 110, member C	0,179
2232	*FDXR*	ferredoxin reductase;AR;adrenodoxin reductase;adrenodoxin-NADP(+) reductase;ferredoxin--NADP(+) reductase	0,147
83888	*FGFBP2*	fibroblast growth factor binding protein 2;37 kDa killer-specific secretory protein;FGF-BP2;FGF-binding protein 2;FGFBP-2;HBp17-RP;HBp17-related protein;killer-specific secretory protein of 37 kDa	0,362
246176	*GAS2L2*	growth arrest-specific 2 like 2;GAS2-related protein on chromosome 17	-0,162
257144	*GCSAM*	germinal center-associated, signaling and motility;germinal center B-cell-expressed transcript 2 protein;germinal center expressed transcript 2;germinal center-associated lymphoma protein;human germinal center-associated lymphoma	0,147
2708	*GJB2*	gap junction protein, beta 2, 26kDa;connexin 26;gap junction protein beta 2;mutant gap junction protein beta 2	0,702
10804	*GJB6*	gap junction protein, beta 6, 30kDa;connexin 30;connexin-30;ectodermal dysplasia 2, hidrotic (Clouston syndrome);gap junction protein, beta 6 (connexin 30)	0,528
2740	*GLP1R*	glucagon-like peptide 1 receptor;GLP-1 receptor;GLP-1-R;GLP-1R	-0,145
2838	*GPR15*	G protein-coupled receptor 15;brother of Bonzo	0,171
84636	*GPR174*	G protein-coupled receptor 174	-0,154
9290	*GPR55*	G protein-coupled receptor 55	0,228
3003	*GZMK*	granzyme K (granzyme 3; tryptase II);NK-Tryp-2;NK-tryptase-2;fragmentin-3;granzyme 3;granzyme K (serine protease, granzyme 3;granzyme-3;tryptase II	-0,155
3090	*HIC1*	hypermethylated in cancer 1;zinc finger and BTB domain-containing protein 29	0,210
79618	*HMBOX1*	homeobox containing 1;homeobox telomere-binding protein 1;homeobox-containing protein PBHNF	-0,149

8870	*IER3*	immediate early response 3;PACAP-responsive gene 1 protein;anti-death protein;differentiation-dependent gene 2 protein;expressed in pancreatic carcinoma;gly96, mouse, homolog of;immediate early protein GLY96;immediate early response 3 protein;immediately early gene X-1;protein DIF-2	0,181
10561	*IFI44*	interferon-induced protein 44;TBC/LysM-associated domain containing 5;interferon-induced, hepatitis C-associated microtubular aggregate protein (44kD);microtubule-associated protein 44	-0,322
10964	*IFI44L*	interferon-induced protein 44-like	-0,451
64135	*IFIH1*	interferon induced with helicase C domain 1;CADM-140 autoantigen;DEAD/H (Asp-Glu-Ala-Asp/His) box polypeptide;RIG-I-like receptor 2;RNA helicase-DEAD box protein 116;clinically amyopathic dermatomyositis autoantigen 140 kDa;helicard;helicase with 2 CARD domains;melanoma differentiation associated protein-5;melanoma differentiation-associated protein 5;murabutide down-regulated protein	-0,160
3433	*IFIT2*	interferon-induced protein with tetratricopeptide repeats 2;Interferon, alpha-inducible protein (MW 54kD);interferon-induced 54 kDa protein;interferon-induced protein 54	-0,267
3437	*IFIT3*	interferon-induced protein with tetratricopeptide repeats 3;CIG49;IFI-60K;IFIT-3;IFIT-4;ISG-60;interferon-induced 60 kDa protein;interferon-induced protein with tetratricopeptide repeats 4;retinoic acid-induced gene G protein	-0,163
50616	*IL22*	interleukin 22;IL-10-related T-cell-derived inducible factor;IL-10-related T-cell-derived-inducible factor;cytokine Zcyto18	0,215
3575	*IL7R*	interleukin 7 receptor;CD127 antigen;IL-7 receptor subunit alpha;IL-7R subunit alpha;IL-7RA;interleukin 7 receptor alpha chain;interleukin 7 receptor isoform H5-6	-0,150
3581	*IL9R*	interleukin 9 receptor;IL-9 receptor	0,216
3631	*INPP4A*	inositol polyphosphate-4-phosphatase, type I, 107kDa;inositol polyphosphate-4-phosphatase, type I, 107kD;type I inositol-3,4-bisphosphate 4-phosphatase	0,197
3394	*IRF8*	interferon regulatory factor 8;interferon consensus sequence binding protein 1	-0,220
81168	*ITM2C*	integral membrane protein 2C;BRICHOS domain containing 2C;cerebral protein 14;integral membrane protein 3;transmembrane protein BRI3	0,307
8850	*KAT2B*	K(lysine) acetyltransferase 2B;CREBBP-associated factor;histone acetylase PCAF;histone acetyltransferase PCAF;lysine acetyltransferase 2B;p300/CBP-associated factor	0,196
56479	*KCNQ5*	potassium channel, voltage gated KQT-like subfamily Q, member 5;KQT-like 5;potassium channel protein;potassium channel subunit alpha KvLQT5;potassium voltage-gated channel, KQT-like subfamily, member 5;voltage-gated potassium channel subunit Kv7.5	-0,232
3815	*KIT*	v-kit Hardy-Zuckerman 4 feline sarcoma viral oncogene homolog;p145 c-kit;piebald trait protein;proto-oncogene c-Kit;proto-oncogene tyrosine-protein kinase Kit;soluble KIT variant 1;tyrosine-protein kinase Kit;v-kit Hardy-Zuckerman 4 feline sarcoma viral oncogene-like protein	0,401
144811	*LACC1*	laccase (multicopper oxidoreductase) domain containing 1	0,179
3903	*LAIR1*	leukocyte-associated immunoglobulin-like receptor 1;leukocyte-associated Ig-like receptor 1	0,177
54900	*LAX1*	lymphocyte transmembrane adaptor 1;LAT-like membrane associated protein;linker for activation of x cells;membrane-associated adapter protein LAX	0,138
4000	*LMNA*	lamin A/C;70 kDa lamin;lamin A/C-like 1;prelamin-A/C;renal carcinoma antigen NY-REN-32	0,259
102724748	*LOC102724748*	uncharacterized LOC102724748	-0,147
10161	*LPAR6*	lysophosphatidic acid receptor 6;G-protein coupled purinergic receptor P2Y5;LPA receptor 6;LPA-6;P2Y purinoceptor 5;RB intron encoded G-protein coupled receptor;oleoyl-L-alpha-lysophosphatidic acid receptor;purinergic receptor 5;purinergic receptor P2Y G protein-coupled protein 5;purinergic receptor P2Y, G-protein coupled, 5	0,251
54947	*LPCAT2*	lysophosphatidylcholine acyltransferase 2;1-AGP acyltransferase 11;1-AGPAT 11;1-acylglycerol-3-phosphate O-acyltransferase 11;1-acylglycerophosphocholine O-acyltransferase;1-alkylglycerophosphocholine O-acetyltransferase;LPAAT-alpha;LPC acyltransferase 2;LPCAT-2;acetyl-CoA:lyso-PAF acetyltransferase;acetyl-CoA:lyso-platelet-activating factor acetyltransferase;acyl-CoA:lysophosphatidylcholine acyltransferase 2;acyltransferase like 1;acyltransferase-like 1;lyso-PAF acetyltransferase;lyso-platelet-activating factor (PAF)	0,204
9404	*LPXN*	leupaxin	0,231
4041	*LRP5*	low density lipoprotein receptor-related protein 5;low density lipoprotein receptor-related protein 7	0,140

2615	*LRRC32*	leucine rich repeat containing 32;garpin;glycoprotein A repetitions predominant	-0,159
54674	*LRRN3*	leucine rich repeat neuronal 3;fibronectin type III, immunoglobulin and leucine rich repeat domains 5;leucine-rich repeat protein, neuronal 3;neuronal leucine-rich repeat protein 3	0,142
1130	*LYST*	lysosomal trafficking regulator;Chediak-Higashi syndrome 1;beige homolog	-0,282
84441	*MAML2*	mastermind-like 2 (Drosophila);mam-2	-0,159
79648	*MCPH1*	microcephalin 1;BRCT-repeat inhibitor of TERT expression 1	-0,319
126308	*MOB3A*	MOB kinase activator 3A;MOB LAK;MOB-LAK;MOB1, Mps One Binder kinase activator-like 2A;mob1 homolog 2A;mps one binder kinase activator-like 2A	0,165
4599	*MX1*	MX dynamin-like GTPase 1;interferon-induced protein p78;interferon-inducible protein p78;interferon-regulated resistance GTP-binding protein MxA;myxoma resistance protein 1;myxovirus (influenza virus) resistance 1, interferon-inducible protein p78	-0,282
4628	*MYH10*	myosin, heavy chain 10, non-muscle;cellular myosin heavy chain, type B;myosin heavy chain, nonmuscle type B;myosin, heavy polypeptide 10, non-muscle;nonmuscle myosin II heavy chain-B;nonmuscle myosin heavy chain 10;nonmuscle myosin heavy chain IIB	0,144
10398	*MYL9*	myosin, light chain 9, regulatory;20 kDa myosin light chain;myosin RLC;myosin regulatory light chain 1;myosin regulatory light chain 2, smooth muscle isoform;myosin regulatory light chain 9;myosin regulatory light chain MRLC1;myosin, light polypeptide 9, regulatory	0,282
64005	*MYO1G*	myosin IG;minor histocompatibility antigen HA-2;myosin-Ig	0,190
26509	*MYOF*	myoferlin;fer-1-like 3, myoferlin;fer-1-like family member 3;fer-1-like protein 3	0,296
85409	*NKD2*	naked cuticle homolog 2 (Drosophila);Dvl-binding protein NKD2	0,244
9111	*NMI*	N-myc (and STAT) interactor;N-myc interactor	-0,163
1728	*NQO1*	NAD(P)H dehydrogenase, quinone 1;DT-diaphorase;NAD(P)H:Quinone acceptor oxidoreductase type 1;NAD(P)H:menadione oxidoreductase 1;NAD(P)H:quinone oxidoreductase 1;NAD(P)H:quinone oxireductase;azoreductase;diaphorase (NADH/NADPH) (cytochrome b-5 reductase);diaphorase-4;dioxin-inducible 1;menadione reductase;phylloquinone reductase;quinone reductase 1	0,181
26012	*NSMF*	NMDA receptor synaptonuclear signaling and neuronal migration factor;nasal embryonic LHRH factor;nasal embryonic luteinizing hormone-releasing hormone factor	0,192
4938	*OAS1*	2'-5'-oligoadenylate synthetase 1, 40/46kDa;(2-5')oligo(A) synthase 1;(2-5')oligo(A) synthetase 1;2',5'-oligo A synthetase 1;2',5'-oligoadenylate synthetase 1, 40/46kDa;2'-5' oligoadenylate synthetase 1 p41 isoform;2'-5' oligoadenylate synthetase 1 p48 isoform;2'-5' oligoadenylate synthetase 1 p49 isoform;2'-5' oligoadenylate synthetase 1 p52 isoform;2'-5'-oligoisoadenylate synthetase 1;2-5A synthase 1;2-5A synthetase 1;E18/E16;p46/p42 OAS	-0,181
8638	*OASL*	2'-5'-oligoadenylate synthetase-like;2'-5'-OAS-RP;2'-5'-OAS-related protein;59 kDa 2'-5'-oligoadenylate synthase-like protein;59 kDa 2'-5'-oligoadenylate synthetase-like protein;TR-interacting protein 14;thyroid receptor-interacting protein 14	-0,269
167826	*OLIG3*	oligodendrocyte transcription factor 3;class B basic helix-loop-helix protein 7;class E basic helix-loop-helix protein 20;oligo3	0,484
5008	*OSM*	oncostatin M	-0,202
5025	*P2RX4*	purinergic receptor P2X, ligand gated ion channel, 4;ATP receptor;ATP-gated cation channel protein;P2X receptor, subunit 4;purinergic receptor P2X, ligand-gated ion channel, 4;purinergic receptor P2X4;purinoceptor P2X4	0,248
64761	*PARP12*	poly (ADP-ribose) polymerase family, member 12;ADP-ribosyltransferase diphtheria toxin-like 12;zinc finger CCCH type domain containing 1	-0,185
83666	*PARP9*	poly (ADP-ribose) polymerase family, member 9;ADP-ribosyltransferase diphtheria toxin-like 9;PARP-9;b aggressive lymphoma protein;poly (ADP-ribose) polymerase 9	-0,161
10769	*PLK2*	polo-like kinase 2;PLK-2;serine/threonine-protein kinase SNK;serum-inducible kinase	0,142
5450	*POU2AF1*	POU class 2 associating factor 1;B-cell-specific coactivator OBF-1;BOB-1;OCA-B;OCT-binding factor 1;POU domain, class 2, associating factor 1	-0,178

5468	*PPARG*	peroxisome proliferator-activated receptor gamma;PPAR-gamma;nuclear receptor subfamily 1 group C member 3;peroxisome proliferator-activated nuclear receptor gamma variant 1	-0,167
81706	*PPP1R14C*	protein phosphatase 1, regulatory (inhibitor) subunit 14C;PKC-potentiated PP1 inhibitory protein;kinase C-enhanced PP1 inhibitor;kinase-enhanced PP1 inhibitor;serologically defined breast cancer antigen NY-BR-81	0,224
10216	*PRG4*	proteoglycan 4;articular superficial zone protein;hemangiopoietin;lubricin;megakaryocyte stimulating factor;superficial zone proteoglycan	-0,266
138639	*PTPDC1*	protein tyrosine phosphatase domain containing 1;protein tyrosine phosphatase PTP9Q22	0,164
5774	*PTPN3*	protein tyrosine phosphatase, non-receptor type 3;cytoskeletal-associated protein tyrosine phosphatase;protein-tyrosine phosphatase H1	0,349
23682	*RAB38*	RAB38, member RAS oncogene family;Rab-related GTP-binding protein;melanoma antigen NY-MEL-1	0,328
5920	*RARRES3*	retinoic acid receptor responder (tazarotene induced) 3;HRAS-like suppressor 4;RAR-responsive protein TIG3;retinoic acid-inducible gene 1;retinoid-inducible gene 1 protein;tazarotene-induced gene 3 protein	-0,179
115727	*RASGRP4*	RAS guanyl releasing protein 4;guanyl nucleotide releasing protein 4	0,174
8787	*RGS9*	regulator of G-protein signaling 9;regulator of G-protein signalling 9	-0,166
9781	*RNF144A*	ring finger protein 144A;UbcM4-interacting protein 4;probable E3 ubiquitin-protein ligase RNF144A;ring finger protein 144;ubiquitin conjugating enzyme 7 interacting protein 4;ubiquitin-conjugating enzyme 7-interacting protein 4	0,149
50484	*RRM2B*	ribonucleotide reductase M2 B (TP53 inducible);TP53-inducible ribonucleotide reductase M2 B;p53-inducible ribonucleotide reductase small subunit 2 homolog;p53-inducible ribonucleotide reductase small subunit 2 short form beta;p53-inducible ribonucleotide reductase small subunit 2-like protein	0,247
1901	*S1PR1*	sphingosine-1-phosphate receptor 1;S1P receptor 1;S1P receptor Edg-1;endothelial differentiation G-protein coupled receptor 1;endothelial differentiation, sphingolipid G-protein-coupled receptor, 1;sphingosine 1-phosphate receptor EDG1;sphingosine 1-phosphate receptor Edg-1	0,141
154075	*SAMD3*	sterile alpha motif domain containing 3;SAM domain-containing protein 3	-0,187
64092	*SAMSN1*	SAM domain, SH3 domain and nuclear localization signals 1;SAM and SH3 domain containing 2;SAM domain, SH3 domain and nuclear localisation signals, 1;SAM domain, SH3 domain and nuclear localization signals protein 1;SH3-SAM adaptor protein;Src homology domain 3 (SH3)-containing adapter protein SH3 lymphocyte protein 2;hematopoietic adapter-containing SH3 and sterile α-motif (SAM) domains 1;hematopoietic adapter-containing SH3 and sterile alpha-motif (SAM) domains 1;hematopoietic adaptor containing SH3 and SAM domains	0,177
6303	*SAT1*	spermidine/spermine N1-acetyltransferase 1;diamine N-acetyltransferase 1;polyamine N-acetyltransferase 1;putrescine acetyltransferase;spermidine/spermine N1-acetyltransferase alpha	-0,144
6304	*SATB1*	SATB homeobox 1;special AT-rich sequence binding protein 1 (binds to nuclear matrix/scaffold-associating DNA);special AT-rich sequence-binding protein 1	-0,194
6402	*SELL*	selectin L;CD62 antigen-like family member L;gp90-MEL;leukocyte surface antigen Leu-8;leukocyte-endothelial cell adhesion molecule 1;lymph node homing receptor;lymphocyte adhesion molecule 1;pln homing receptor	-0,233
27244	*SESN1*	sestrin 1;p53 activated gene 26;p53 regulated PA26 nuclear protein	0,151
57823	*SLAMF7*	SLAM family member 7;19A24 protein;CD2 subset 1;CD2-like receptor activating cytotoxic cells;CD2-like receptor-activating cytotoxic cells;membrane protein FOAP-12;novel LY9 (lymphocyte antigen 9) like protein;protein 19A	0,160
6509	*SLC1A4*	solute carrier family 1 (glutamate/neutral amino acid transporter), member 4;ASCT-1;alanine/serine/cysteine/threonine transporter 1;glutamate/neutral amino acid transporter;solute carrier family 1 member 4	0,146
51312	*SLC25A37*	solute carrier family 25 (mitochondrial iron transporter), member 37;mitochondrial iron transporter 1;mitochondrial solute carrier protein;mitoferrin;predicted protein of HQ2217;solute carrier family 25, member 37	-0,159
5172	*SLC26A4*	solute carrier family 26 (anion exchanger), member 4;sodium-independent chloride/iodide transporter	-0,173
6513	*SLC2A1*	solute carrier family 2 (facilitated glucose transporter), member 1;choreoathetosis/spasticity, episodic (paroxysmal choreoathetosis/spasticity);glucose transporter type 1, erythrocyte/brain;hepG2 glucose transporter;human T-cell leukemia virus (I and II) receptor;receptor for HTLV-1 and HTLV-2	0,164
401548	*SNX30*	sorting nexin family member 30	0,149

6654	*SOS1*	son of sevenless homolog 1 (Drosophila);SOS-1;gingival fibromatosis, hereditary, 1;guanine nucleotide exchange factor	-0,143
401236	*STMND1*	stathmin domain containing 1	0,236
387082	*SUMO4*	small ubiquitin-like modifier 4;SMT3 suppressor of mif two 3 homolog 2;SMT3 suppressor of mif two 3 homolog 4;insulin-dependent diabetes mellitus 5;small ubiquitin-like modifier 4 protein	0,302
7041	*TGFB1I1*	transforming growth factor beta 1 induced transcript 1;androgen receptor coactivator 55 kDa protein;androgen receptor coactivator ARA55;androgen receptor-associated protein of 55 kDa;hydrogen peroxide-inducible clone 5 protein;hydrogen peroxide-inducible clone-5	0,145
7070	*THY1*	Thy-1 cell surface antigen;CDw90;Thy-1 T-cell antigen;thy-1 antigen	-0,142
201633	*TIGIT*	T cell immunoreceptor with Ig and ITIM domains;V-set and immunoglobulin domain containing 9;V-set and immunoglobulin domain-containing protein 9;V-set and transmembrane domain containing 3;V-set and transmembrane domain-containing protein 3;Washington University cell adhesion molecule	-0,265
25976	*TIPARP*	TCDD-inducible poly(ADP-ribose) polymerase;ADP-ribosyltransferase diphtheria toxin-like 14;poly [ADP-ribose] polymerase 7	0,204
51768	*TM7SF3*	transmembrane 7 superfamily member 3;seven span transmembrane protein;seven transmembrane protein TM7SF3	0,528
153339	*TMEM167A*	transmembrane protein 167A;transmembrane protein 167	0,144
23670	*TMEM2*	transmembrane protein 2	0,491
9540	*TP53I3*	tumor protein p53 inducible protein 3;p53-induced gene 3 protein;quinone oxidoreductase homolog	0,186
9618	*TRAF4*	TNF receptor-associated factor 4;MLN 62;RING finger protein 83;TRAF4 variant 6;cysteine-rich domain associated with RING and Traf domains protein 1;cysteine-rich domain associated with ring and TRAF domain;malignant 62;metastatic lymph node gene 62 protein;tumor necrosis receptor-associated factor 4A	0,140
1831	*TSC22D3*	TSC22 domain family, member 3;DSIP-immunoreactive leucine zipper protein;DSIP-immunoreactive peptide;TSC-22 related protein;TSC-22-like protein;TSC-22-related protein;delta sleep inducing peptide, immunoreactor;delta sleep-inducing peptide immunoreactor;glucocorticoid-induced leucine zipper protein	0,146
90139	*TSPAN18*	tetraspanin 18;tspan-18	-0,173
10628	*TXNIP*	thioredoxin interacting protein;thioredoxin binding protein 2;thioredoxin-binding protein 2;upregulated by 1,25-dihydroxyvitamin D-3;vitamin D3 up-regulated protein 1	0,149
401447	*USP17L1*	ubiquitin specific peptidase 17-like family member 1;deubiquitinating enzyme 17-like protein 1;putative ubiquitin carboxyl-terminal hydrolase 17-like protein 1;ubiquitin thioesterase 17-like protein 1;ubiquitin-specific-processing protease 17-like protein 1	0,141
64393	*ZMAT3*	zinc finger, matrin-type 3;WIG-1/PAG608 protein;p53 target zinc finger protein;p53-activated gene 608 protein;zinc finger protein WIG-1;zinc finger protein WIG1;zinc finger, matrin type 3	0,199
